# Supplementary material for: Transcriptome Analysis Reveals the Molecular Mechanism and Responsive Genes of Waterlogging Stress in Actinidia deliciosa Planch Kiwifruit Plants
Source: Int J Mol Sci. 2023 Nov 1;24(21):15887. doi: 10.3390/ijms242115887 (PMC10649176; doi:10.3390/ijms242115887)
Supplement: Supplementary file 1 [file ijms-24-15887-s001.zip › ijms-2648385-supplementary.pdf]

Figure S1. The numbers of total transcripts and new annotated transcripts.

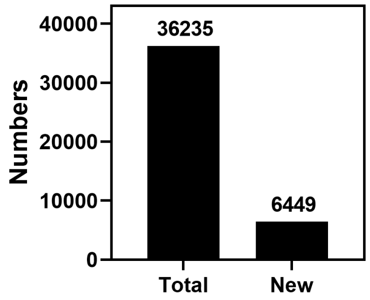

Table S1. The statistics of RNA-seq.

Sample	Clean reads	Clean bases	GC Content	Q20 (%)	Q30 (%)
W0dA	20,148,045	6,030,832,210	46.40%	97.78%	93.70%
W0dB	22,857,104	6,839,556,026	46.29%	97.41%	92.94%
W0dC	20,228,146	6,054,052,424	47.06%	97.51%	93.19%
W3dA	22,048,322	6,598,447,368	46.47%	97.68%	93.51%
W3dB	21,408,074	6,407,554,854	46.52%	97.70%	93.54%
W3dC	22,712,855	6,797,201,834	46.42%	97.43%	92.96%
W7dA	20,083,211	6,010,383,562	46.02%	97.90%	94.00%
W7dB	20,759,920	6,212,575,174	46.13%	97.80%	93.78%
W7dC	22,516,992	6,740,252,426	45.92%	97.30%	92.78%

Table S2. Statistical results of sequence alignment between the sample reads and the reference genome.

Sample	Total Reads	Mapped Reads	Uniq Mapped Reads	Multiple Map Reads
W0dA	40,296,090	33,341,967 (82.74%)	31,385,221 (77.89%)	1,956,746 (4.86%)
W0dB	45,714,208	37,555,373 (82.15%)	35,574,405 (77.82%)	1,980,968 (4.33%)
W0dC	40,456,292	33,441,629 (82.66%)	29,291,720 (72.40%)	4,149,909 (10.26%)
W3dA	44,096,644	36,068,031 (81.79%)	33,835,270 (76.73%)	2,232,761 (5.06%)
W3dB	42,816,148	34,993,853 (81.73%)	32,739,251 (76.46%)	2,254,602 (5.27%)
W3dC	45,425,710	37,004,558 (81.46%)	34,527,019 (76.01%)	2,477,539 (5.45%)
W7dA	40,166,422	31,947,505 (79.54%)	30,440,591 (75.79%)	1,506,914 (3.75%)
W7dB	41,519,840	33,031,589 (79.56%)	31,219,312 (75.19%)	1,812,277 (4.36%)
W7dC	45,033,984	35,788,781 (79.47%)	34,134,696 (75.80%)	1,654,085 (3.67%)

Table S3. The detailed information of DEGs classified significantly by GO enrichment.

Samples	GO Term	GO class	Number of enriched DEGs	Total annotated DEGs by GO
W0d/W3d	oxidoreductase activity	4	114	8139
	plasma membrane	4	75	
	UDP-glycosyltransferase activity	6	75	
	integral component of membrane	5	1132	
	protein phosphatase inhibitor activity	7	24	
W0d/W7d	monooxygenase activity	5	116	7570
	oxidoreductase activity	4	112	
	protein phosphatase inhibitor activity	7	25	
	UDP-glycosyltransferase activity	6	70	
	protein kinase activity	7	320	
	photosystem I	7	24	
W3d/W7d	photosystem I	7	29	4900
	photosystem I reaction center	6	10	
	hydrolase activity, hydrolyzing O-glycosyl compounds	6	73	
	extracellular region	3	35	
	monooxygenase activity	5	77	
	oxidoreductase activity	4	73	
	UDP-glycosyltransferase activity	6	48	

Table S4. The expression information of DEGs related to oxidoreductase activity.

Genes	W0dA	W0dB	W0dC	W3dA	W3dB	W3dC	W7dA	W7dB	W7dC
Acc11493	4.47	3.46	3.36	9.87	8.19	7.92	0.15	0.51	0.85
Acc19879	6.81	6.25	7.13	40.04	42.50	41.34	21.49	23.17	22.81
Acc27815	186.24	184.09	183.94	12.13	11.66	11.96	43.06	45.65	45.23
Acc27999	0.72	0.45	0.44	4.06	4.86	4.90	1.82	1.53	1.44
Acc25259	0.19	0.13	0.04	2.55	3.11	2.95	1.19	0.70	0.70
Acc01793	143.25	149.56	144.27	0.29	0.53	0.36	6.86	7.37	4.79
Acc00220	9.61	9.35	9.94	32.36	28.48	30.90	2.62	3.20	2.63
Acc23331	0.16	0.45	0.34	1.11	1.72	1.51	4.20	5.18	4.33
Acc25077	6.25	5.79	4.86	0.10	0.15	0.28	0.93	0.68	0.85
Acc00668	0.27	0.36	0.00	1.11	1.43	1.20	4.22	3.14	3.76
Acc30033	3.73	4.22	4.92	1.33	2.24	1.71	0.73	0.74	0.55
Acc16462	124.77	123.84	124.04	721.70	719.43	731.22	284.94	280.02	291.53
Acc01958	16.73	15.90	15.02	31.97	34.34	29.57	83.60	77.76	76.09
Acc13383	2.87	2.24	2.60	0.59	1.21	0.41	8.85	6.66	7.40
Acc08451	5.21	6.23	5.42	33.27	31.95	34.27	0.71	0.71	0.19
Acc20538	1.36	1.42	2.03	6.18	6.94	5.61	34.12	32.85	32.40
Acc28739	24.21	26.22	27.44	2.33	1.61	1.48	6.81	6.21	8.01
Acc28738	55.19	59.05	49.12	3.47	4.14	4.06	16.30	15.37	17.39

Acc06792	22.53	20.86	20.68	2.13	2.37	2.18	8.32	5.32	7.23
Acc23323	47.36	41.62	38.58	2007.71	1948.40	1958.77	18.64	17.59	17.61
Acc06537	92.47	91.68	89.60	14.32	15.89	15.20	36.20	38.22	38.63
Acc29858	0.92	1.80	1.71	8.42	7.56	7.92	3.02	3.22	4.59
Acc24093	1.82	2.10	2.00	23.36	23.81	24.51	6.32	3.65	4.10
Acc13003	0.26	0.47	0.58	2.12	3.37	3.55	8.11	5.36	7.92

Table S5. The expression information of DEGs related to UDP-glycosyltransferase activity.

Genes	W0dA	W0dB	W0dC	W3dA	W3dB	W3dC	W7dA	W7dB	W7dC
Acc15775	12.06	14.86	13.99	1.29	1.31	1.17	4.19	4.14	3.96
Acc26546	1.16	1.63	2.02	2.44	4.61	3.49	29.44	27.97	28.97
Acc26545	3.35	3.79	4.69	18.64	21.86	22.97	12.88	11.23	11.41
Acc02866	0.81	1.42	1.96	0.64	0.28	0.74	3.04	4.18	4.33
Acc25791	6.20	6.96	5.11	0.03	0.00	0.12	1.44	3.00	2.55
Acc08454	2.09	2.14	2.17	30.11	26.80	26.50	11.85	8.98	10.57
Acc01281	0.28	0.34	0.24	5.35	5.70	4.40	1.67	1.09	1.22
Acc03120	0.78	0.90	0.64	1.66	2.00	1.56	4.88	4.71	4.95
Acc08453	2.58	2.07	2.35	41.93	41.42	38.94	5.45	5.33	5.20
Acc27769	1.52	2.59	2.01	4.35	4.68	5.97	12.83	15.63	14.89
Acc27565	0.53	0.56	0.64	2.33	3.33	3.13	12.26	10.67	10.55
Acc21186	110.33	111.19	108.14	6.43	7.98	9.70	32.31	33.01	35.73
Acc30749	0.23	0.25	0.22	2.08	2.17	2.52	0.92	1.08	1.43
Acc06935	5.06	5.87	6.00	66.26	73.86	64.72	26.54	27.53	25.20
Acc30748	0.55	0.71	0.60	5.92	7.44	7.79	3.62	3.49	4.55
Acc10216	57.78	54.80	51.98	0.07	0.00	0.13	3.84	4.40	3.68
Acc19983	0.56	0.59	0.52	6.04	7.03	6.40	3.53	3.08	4.57

Table S6. The information of screened candidate genes. The ADH genes were marked in red, and transcription factors were marked in blue.

Genes	Log_2FC	W0d/W3d	Log_2FC	W0d/W7d	Log_2FC	W3d/W7d	Nr annotation
Acc10216	-9.53	down	-3.96	down	5.54	up	zeatin O-glucosyltransferase-like [*Nicotiana attenuata*]
Acc09373	1.87	up	3.48	up	1.57	up	PREDICTED: glutathione S-transferase [*Vitis vinifera*]
Acc11090	1.62	up	3.26	up	1.60	up	dormancy-associated protein homolog 3-like isoform X2 [*Cajanus cajan*]
Acc04770	3.35	up	4.66	up	1.27	up	PREDICTED: subtilisin-like protease SBT1.7 [*Vitis vinifera*]
Acc07008	4.50	up	5.77	up	1.24	up	uncharacterized protein LOC18767295 [*Prunus persica*]
Acc16985	-1.91	down	-5.09	down	-3.21	down	PREDICTED: WUSCHEL-related homeobox 11 isoform X1 [*Ricinus communis*]
Acc12148	-1.11	down	-2.57	down	-1.50	down	PREDICTED: AT-hook motif nuclear-localized protein 17-like [*Ziziphus jujuba*]
Acc03015	-1.58	down	-2.78	down	-1.23	down	abscisic acid receptor PYL4-like [*Hevea brasiliensis*]
Acc31645	-1.24	down	-2.24	down	-1.04	down	GA signaling receptor [Actinidia deliciosa]
Acc11677	-1.91	down	-2.91	down	-1.03	down	Histidine-rich glycoprotein [*Actinidia chinensis* var. chinensis]
Acc15356	-3.43	down	-4.58	down	-1.19	down	zinc transporter 1 [*Prunus persica*]
Acc14256	-1.07	down	-3.15	down	-2.11	down	PREDICTED: vinorine synthase [*Theobroma cacao*]
Acc04291	-1.51	down	-3.52	down	-2.05	down	sugar transport protein 13 [*Jatropha curcas*]
Acc28092	-1.50	down	-3.59	down	-2.13	down	Expansin-related protein 1 precursor [*Populus trichocarpa*]
Acc17117	-1.59	down	-3.88	down	-2.33	down	uncharacterized protein LOC110229480 [*Arabidopsis lyrata subsp.* lyrata]
Acc23514	-2.15	down	-3.99	down	-1.88	down	alpha-amylase [*Actinidia chinensis*]

Acc01853	-1.07	down	-2.87	down	-1.84	down	PREDICTED: cationic peroxidase 1 [*Vitis vinifera*]
Acc23194	-1.29	down	-2.61	down	-1.37	down	hypothetical protein CDL15_Pgr003127 [*Punica granatum*]
Acc10167	-2.13	down	-3.88	down	-1.79	down	PREDICTED: uncharacterized protein LOC109011949 [*Juglans regia*]
Acc29514	-1.14	down	-3.86	down	-2.76	down	vacuolar-processing enzyme [*Jatropha curcas*]
Acc01720	-1.21	down	-2.90	down	-1.73	down	PREDICTED: protein SSUH2 homolog [*Ricinus communis*]
Acc25087	-1.88	down	-2.93	down	-1.08	down	polyphenoloxidase [*Camellia nitidissima*]
Acc15162	-1.26	down	-2.39	down	-1.18	down	BI1-like protein [*Prunus persica*]
Acc30340	-2.04	down	-3.30	down	-1.30	down	PREDICTED: uncharacterized protein LOC18589995 [*Theobroma cacao*]
Acc14629	-1.11	down	-2.36	down	-1.29	down	PREDICTED: myb-related protein Myb4-like [*Gossypium hirsutum*]
Acc28506	-2.54	down	-8.35	down	-5.85	down	RBCS1 [*Actinidia chinensis*]
Acc28101	-2.07	down	-4.00	down	-1.97	down	Barwin-like endoglucanase [*Cynara cardunculus* var. scolymus]
Acc12842	-1.79	down	-2.88	down	-1.13	down	PREDICTED: GDSL esterase/lipase 5 [*Vitis vinifera*]
Acc14982	-1.52	down	-3.10	down	-1.62	down	fatty acid hydroperoxide lyase [*Camellia oleifera*]
Acc27749	3.24	up	-3.10	up	-2.27	down	unnamed protein product, partial [*Vitis vinifera*]
Acc31801	3.13	up	1.00	up	-2.07	down	uncharacterized protein LOC110412173 isoform X2 [*Herrania umbratica*]
Acc05506	9.31	up	1.10	up	-3.69	down	PREDICTED: uncharacterized protein LOC8263583 [*Ricinus communis*]
Acc19764	3.53	up	5.66	up	-2.06	down	PREDICTED: uncharacterized protein LOC100260339 isoform X2 [*Vitis vinifera*]

Acc31448	4.63	up	1.51	up	-2.91	down	probable 6-phosphogluconolactonase 1 [*Herrania umbratica*]
Acc15006	3.30	up	1.76	up	-2.23	down	unnamed protein product, partial [*Vitis vinifera*]
Acc07453	6.77	up	1.11	up	-4.64	down	3-ketoacyl-CoA synthase [*Actinidia chinensis*]
Acc08779	3.90	up	2.18	up	-2.58	down	PREDICTED: cysteine proteinase inhibitor B-like [*Juglans regia*]
Acc11566	3.94	up	1.35	up	-2.61	down	ADH2 [*Actinidia deliciosa*]
Acc19486	5.25	up	1.37	up	-2.65	down	stem-specific protein TSJT1 [*Herrania umbratica*]
Acc04406	3.49	up	2.64	up	-2.32	down	PREDICTED: dynein light chain 2, cytoplasmic [*Solanum tuberosum*]
Acc05326	4.49	up	1.21	up	-2.30	down	PREDICTED: uncharacterized protein LOC104607004 [*Nelumbo nucifera*]
Acc19929	3.78	up	2.22	up	-2.09	down	PREDICTED: uncharacterized protein LOC102580155 [*Solanum tuberosum*]
Acc26216	4.28	up	1.73	up	-2.14	down	F-box protein At2g27310 [*Prunus avium*]
Acc08443	3.60	up	2.18	up	-2.08	down	ESF1 homolog [*Manihot esculenta*]
Acc16908	6.30	up	1.55	up	-4.45	down	transcriptional regulator EFH1 isoform X1 [*Herrania umbratica*]
Acc04509	5.25	up	1.89	up	-3.29	down	PREDICTED: protein GDAP2 homolog [*Nelumbo nucifera*]
Acc08063	5.11	up	2.01	up	-3.48	down	ADH1 [*Actinidia deliciosa*]
Acc25497	3.25	up	1.66	up	-2.08	down	sucrose synthase [*Actinidia deliciosa*]
Acc04162	3.80	up	1.21	up	-2.27	down	Glutathione S-transferase tau 7 isoform 2, partial [*Theobroma cacao*]
Acc23776	3.56	up	1.56	up	-2.10	down	PREDICTED: probable isoaspartyl peptidase/L-asparaginase 2 [*Nicotiana attenuata*]

Table S7. The primer sequences used for quantitative real-time PCR analysis.

Gene	Forward (5' to 3')	Reverse (5' to 3')
AdADH1	CCATCCGATGAATCTCTTGAA	CGAAAGCCTTGTTGATCTCAG
AdADH2	CAAGACACACCCGATGAATTT	CCGAGAACGAGACTTGATGAG
Acc26216	GACGGGAAGAGTTTGTGCTG	CACTAGAGGCAATGCAACCC
Acc08443	GGCGAGTGTGGTTCAAGATG	TAATGGAGGACGGATCGCTC
Acc16908	GTCATTGCAGTTGGAGGAGC	CGGTAATGGAGGACGGATCG
AcACTIN	TGCATGAGCGATCAAGTTTCAAG	CAACGTTGACAACATCAGGC

Table S8. The primer sequences used for cloning AdADH1 and AdADH2 promoters.

Gene	Forward (5' to 3')
AdADH1-FP1	TGCTTTTGAGAAATCCGGTACA
AdADH1-FP2	GATATTGACTACAGTTTTCGGATTC
AdADH1-FP3	CTCTTCAACCTTTGATGAAAATGT
AdADH1-FP4	TCAATTAAACAAATGGAAAAATGG
AdADH1-FP5	GTTTCACCATTCATATTACGCC
AdADH1-RP1	TGCCCAGCAGTGCTTGCCAT
AdADH1-RP2	CTCTCTCTCTCTAACTTCTC
AdADH2-FP1	ATTGAGAAGTCTAATTGTAATATCCCAT
AdADH2-FP2	TGATCGGATACAAATCAATATCCG
AdADH2-FP3	ATCACATATCAGTAAATATATGATCGG
AdADH2-FP4	GGACCTGTTTGGGCATCGCC
AdADH2-FP5	TGGTCCAAAAATATTGTCTACTACAC
AdADH2-FP6	ATATCAAGATAAATTGATATAATCATAT
AdADH2-FP7	AATGAGCCCAAAATTAAACCC
AdADH2-RP1	CTTGACCAGCAGTGCTTGACAT
AdADH2-RP2	CTCTTCCCTTTCCTTCTTCACTGG
